# Ribosome biogenesis‐based predictive biomarkers in endocrine therapy (Anastrozole) combined with mTOR inhibitor (Vistusertib) in endometrial cancer: translational study from the VICTORIA trial in collaboration with the GINECO group

**DOI:** 10.1002/1878-0261.13340

**Published:** 2022-12-07

**Authors:** Nour‐El‐Houda Mourksi, Cécile Dalban, Amélie Colombe‐Vermorel, Laetitia Odeyer, Valentin Simioni, Jean‐Sébastien Frenel, Michel Fabbro, Fernando Bazan, Sophie Abadie‐Lacourtoisie, Elodie Coquan, Séverine Martinez, Gwenaelle Garin, Séverine Tabone‐Eglinger, Isabelle Treilleux, Sylvie Chabaud, David Pérol, Isabelle Ray‐Coquard, Pierre‐Etienne Heudel, Jean‐Jacques Diaz, Virginie Marcel

**Affiliations:** ^1^ Centre de Recherche en Cancérologie de Lyon, Inserm U1052, CNRS UMR5286, Université Claude Bernard Lyon 1, Centre Léon Bérard Université de Lyon Lyon France; ^2^ Institut Convergence PLAsCAN Lyon France; ^3^ DevWeCan Labex Laboratory Lyon France; ^4^ Clinical Research Department Centre Léon Bérard Lyon France; ^5^ Biopathology Department Centre Léon Bérard, GINECO Lyon France; ^6^ Medical Oncology Department, Institut Cancérologie de l'Ouest, and GINEGEPS St Herblain France; ^7^ Department of Surgical Oncology, Institut du Cancer de Montpellier University of Montpellier Montpellier France; ^8^ Department of Medical Oncology University Hospital of Besançon Besançon France; ^9^ Institut de Cancérologie de l'Ouest Angers France; ^10^ Department of Clinical Research, Department of Medical Oncology Comprehensive Cancer Centre François Baclesse Caen France; ^11^ Biological Resource Center, Centre Léon Bérard Lyon France; ^12^ Medical Oncology Department Centre Léon Bérard and University Claude Bernard Lyon 1, GINECO Lyon France

**Keywords:** advanced endometrial cancer, biomarker, clinical trial, endocrine therapy, mTOR inhibitor, ribosome biogenesis

## Abstract

Resistance of advanced hormone‐dependent endometrial carcinoma to endocrine therapy remains a worldwide clinical issue. We recently reported that the combination of Vistusertib (V, mTOR inhibitor) and Anastrozole (A, aromatase inhibitor) improves the progression‐free rate compared to Anastrozole alone. However, a better patient selection based on biomarkers would improve patient outcome. We evaluate for the first time the usage of ribosome biogenesis (RiBi) factors as a source of innovative markers. Using 47 FFPE tumours (A *n* = 18; V + A *n* = 29), 32 blood samples (A *n* = 13; V + A *n* = 19) and 30 samples of total RNAs (A *n* = 12; V + A *n* = 18) from the VICTORIA clinical trial, we observed an association between RiBi‐associated markers and drug activity or prediction of treatment response. *NOP10 and NHP2* mRNA levels were significantly higher in non‐responders compared to responders in the Vistusertib + Anastrozole arm (*P* = 0.0194 and *P* = 0.0002 respectively; i.e. 8 weeks progression‐free survival as endpoint). This study provides RiBi‐based markers relevant for a better selection of patients with advanced endometrial carcinoma by predicting the response of endocrine therapy combined with mTOR inhibitor.

## Graphical abstract

Schematic workflow of the first ancillary research associated to the randomized phase I/II VICTORIA trial (NCT02730923, Anastrozole + Vistusertib in advanced endometrial cancer, Heudel et al. *JAMA Oncol* 2022) that investigates the ribosome biogenesis factors as predictive markers of combined aromatase/mTOR inhibitors. Distinct expression patterns of two RiBi genes *(NHP2* and *NOP10)* were observed in responders and non‐responders of Vistusertib + Anastrozole combination therapy.

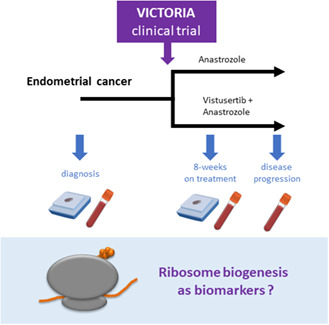

**Abbreviations**
A: Anastrozole
Diag: diagnostic
DKC1: dyskerin pseudouridine synthase 1
FBL: fibrillarin
FFPE: formalin‐fixed, paraffin‐embedded
HR+: hormone receptor positive
IHC: immunohistochemistry
NCL: nucleolin
NHP2: NHP2 ribonucleoprotein
NOP10: NOP10 ribonucleoprotein
On T: on treatment
p4EBP1: phosphorylated 4EBP1
POLR1A: RNA polymerase I subunit A
RiBi: ribosome biogenesis
rRNA: ribosomal RNA
RT: reverse transcription
S6K: S6 kinase
TAF1B: RNA polymerase I subunit B
UCEC: uterine corpus endometrial carcinoma
V: Vistusertib

## 1 Introduction

Endometrial cancer is the most frequent gynaecological cancer worldwide [1]. The survival rate remains poor in advanced disease. The 5‐year survival rate is indeed of only 25% in metastatic patients, mainly due to limited response to systemic therapy [2, 3]. Most of the endometrial tumours are hormone receptor positive (HR+, 60%) and harbour mutations, including in the PI3K/AKT/mTOR pathway (about 40–50%). In HR+ metastatic endometrial patients, although endocrine therapy allows response in 15–30% of cases, it is only of short duration. In order to avoid resistance to endocrine therapy, we recently evaluated safety and efficacy of combining the aromatase inhibitor Anastrozole with the mTOR inhibitor Vistusertib, in 73 patients with advanced or relapsed HR+ endometrial cancer maximally treated with one line of chemotherapy [4]. This multicentric, randomized open‐label phase I/II VICTORIA trial (NCT02730923) showed that Vistusertib and Anastrozole combination treatment (V + A, *n* = 49) improve neoplastic control compared to Anastrozole alone (A, *n* = 24), with a progression‐free rate at 8 weeks of 67% in the V + A arm and of 39% in the A arm. The VICTORIA trial thus reveals that targeting the PI3K/AKT/mTOR pathway is of clinical relevance in metastatic endometrial cancer. Nevertheless, a better selection of patients based on molecular traits of their tumours would improve outcome, by predicting the effectiveness of mTOR inhibitors [4, 5].

Ribosome, the cellular machinery translating mRNA into protein, appears at the heart of several recent innovative approaches to improve cancer patient management [6, 7, 8]. It appears that most cancer cells are addicted to ribosome biogenesis (RiBi). Cancer cells display RiBi hyperactivation to maintain a high rate of protein synthesis required to sustain cell growth and hyperproliferation. Increased RiBi results from several oncogenic activations, including the oestrogen and mTOR signalling pathways [8, 9]. mTORC1 coordinates the multistep process of RiBi through phosphorylation of its two targets, S6 kinase (S6K) and 4EBP1, by promoting ribosomal RNA (rRNA) transcription or expression of factors required for ribosome biogenesis [8, 10]. Moreover, by promoting the phosphorylation of 4EBP1, mTORC1 disrupts the 4EBP1:eIF4E complex thus promoting translational initiation in an eIF4E‐dependent manner in the cytoplasm. Up to date, we and others contributed in demonstrating that several RiBi factors are relevant prognostic biomarkers in different cancer types [7, 11, 12, 13]. However, only few studies have investigated the predictive values of RiBi factors in response to targeted therapies particularly using prospective sample collection. Here, we made the preliminary evaluation of potential RiBi‐based biomarkers in response to aromatase and mTOR inhibitors thanks to the VICTORIA clinical trial.

## 2 Materials and methods

### 2.1 Study design and samples

The randomized phase I/II VICTORIA trial (NCT02730923) was conducted in HR+ metastatic or recurrent endometrial cancer patients (*n* = 73) to evaluate the Vistusertib + Anastrozole combination (V + A) towards Anastrozole alone (A) [4]. Tumour biopsies at diagnosis and after 8 weeks of treatment were stored as formalin‐fixed, paraffin‐embedded (FFPE) blocks before processing at Centre Léon Bérard. Blood samples were collected in EDTA tubes at pre‐treatment, after 8 weeks of treatment and at disease progression. Serum was directly separated by 2000 ***g*** centrifugation for 10 min and stored at −20 °C. Biological samples have been prepared by BB‐0033‐00050, CRB Centre Léon Bérard, Lyon France. It has to be noted that our cohort is limited in terms of sample numbers. First, the study was designed as a two steps trial and the optimal (and minimal) cohort size has been defined according to the Simon optimal two‐stage design. Thus, a total of 73 patients have been enrolled in the study so that the number of patients is relevant for the study (24 patients in the Anastrozole arm vs. 49 patients in the Anastrozole + Vistusertib arm). Among the 73 enrolled patients, material samples were lacking for some patients, particularly regarding the 8 weeks on treatment biopsies since most of the patients progressed during the course of the trial avoiding performing additional biopsies. Moreover, most of the biopsies corresponded to small tumours providing thus insufficient quantity of material to perform both immunohistochemistry and molecular analyses. In these cases, priority was given to immunohistochemistry.

### 2.2 Immunohistochemistry

Immunohistochemical (IHC) staining was performed on 4‐μm thick sections of FFPE tumours. Using the Benchmark Ultra automate (Ventana Medical System, Oro Valley, AZ, USA), several markers were analysed: nuclear 4EBP1 (total: ref#9644; phosphorylated: ref#4857; Cell Signaling Technology, Danvers, MA, USA); cytoplasmic S6K (total: ref#2317; phosphorylated: ref#4857; Cell Signaling Technology, Danvers, MA, USA); nuclear and nucleolar NCL (ADI‐KAM‐CP100; Enzo Life Sciences, Ann Arbor, MI, USA); and nucleolar FBL (ab154806; Abcam, Cambridge, UK). Detection was performed using the ultraView Universal DAB Detection kit (Roche, Basel, Switzerland). Slide reading was performed by anatomopathologist and IHC scores were determined for each marker (Table S1).

### 2.3 Medium throughput RTqPCR

Tumoural tissues from FFPE blocks were identified and cellular content estimated by anatomopathologist prior to total RNA purification. Total RNA was extracted using the Allprep DNA/RNA kit (Qiagen, Venlo, Netherlands). RNA concentration and absorbance were analysed with NanoDrop spectrophotometer (Thermo Fisher Scientific, Waltham, MA, USA), and quality profiles assessed on Tapestation (Agilent Technologies, Santa Clara, CA, USA). mRNA levels of 20 RiBi genes were quantified by medium throughput RTqPCR using the Biomark HD system (Fluidigm, South San Francisco, CA, USA) as previously described [13]. Reverse transcription (RT) was performed on 200 ng of total RNA using the Prime Script RT Reagent kit (Takara, Tokyo, Japan). After multiplex PCR and Exonuclease I treatment, qPCR was carried out on a 96.96 Dynamic Array IFC (Fluidigm, South San Francisco, CA, USA) using the Master Mix 2X EvaGreen (Biorad, Hercules, CA, USA; Table S2). Each sample was analysed using two independent RT and each RT was quantified in triplicate. Human Xpress Ref Universal Total RNA (Qiagen, Venlo, Netherlands) and the median Ct value of five housekeeping genes were used as normalizers.

### 2.4 ELISA

Detection of both circulating FBL peptides and anti‐FBL autoantibodies in serum was performed using the ELISA kits OKWB00172 (dilution 1 : 2) and OKCA00416 (dilution 1 : 101) respectively (Aviva Systems Biology, San Diego, CA, USA). A standard curve using four serial dilutions of the provided positive control was used for quantification.

### 2.5 Statistical analysis

Descriptive statistics were used to summarize patient characteristics. Comparison between two unpaired groups was performed using Wilcoxon–Mann–Whitney non‐parametric test. Significance was considered for two‐tailed *P*‐value < 0.05. In VICTORIA clinical trial, 8 weeks progression‐free survival was used as endpoint. Thus, responders correspond to patients with no progression during the first 8 weeks of treatment and non‐responders to patients who progressed during the first 8 weeks of treatment. Statistics were performed using sas version 9.4 (SAS Institute, Cary, NC, USA). Graphical representations were performed using graphpad prism version 8.0.1 (GraphPad Software, San Diego, CA, USA). Overall survival was performed using the web portal UALCAN, which performs Kaplan–Meier survival analysis using TCGA datasets (UCEC: Uterine Corpus Endometrial Carcinoma) and quartile as a cut‐off (high: 25% of the tumours expressing the highest level of the gene of interest; low: 75% of the remaining tumours) [14].

### 2.6 Ethical approval

All the experiment protocols for involving humans were in accordance with the guidelines of national and institutional entities based on the Declaration of Helsinki in the manuscript. All experimental protocols were approved by the French Competent Authority (ANSM) and by an independent French Ethic Committee (CPP Sud Est IV). Written informed consent was obtained from all patients at their inclusion.

## 3 Results

### 3.1 Protein detection of RiBi factors according to aromatase and mTOR inhibitors treatment

**Fig. 1:**
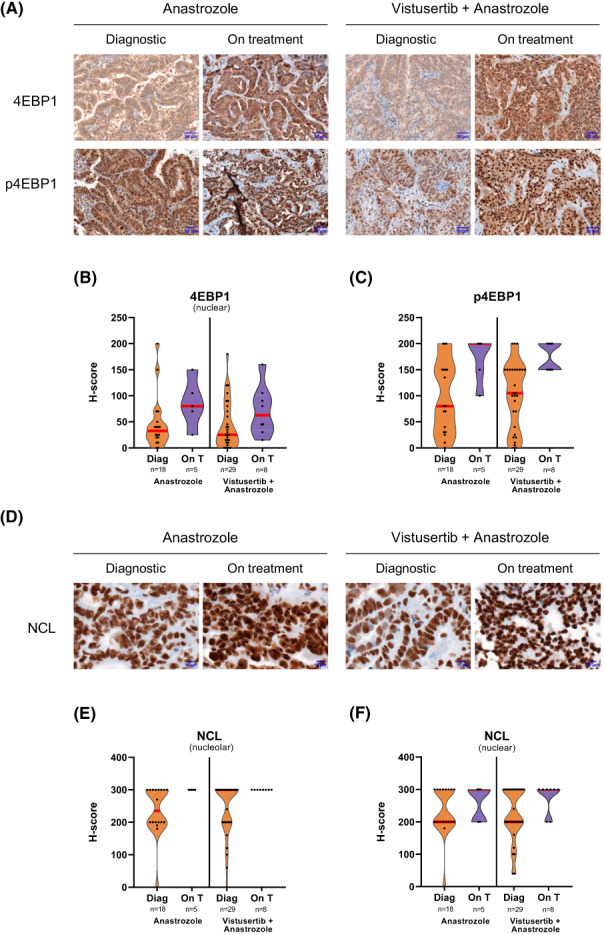
Changes of 4EBP1 and NCL staining according to aromatase and mTOR inhibitors treatment. Detection of total and phosphorylated 4EBP1 marker (4EBP1 and p4EBP1 respectively) (A–C) and of nucleolar and nuclear nucleolin (NCL) marker (D–F) was compared using immunohistochemistry (IHC) between the two arms (A: Anastrozole; V + A: Vistusertib + Anastrozole) and during the course of the patient treatment (Diag: Diagnostic; vs. on T: 8 weeks on treatment). A representative IHC is given among the four groups for 4EBP1 (A, magnification: 30×, scale bar: 50 μm) and NCL (D, magnification: 90, scale bar: 10 μm). H‐scores of total (B) and phosphorylated 4EBP1 (C), and nucleolar (E) and nuclear NCL (F) stainings are represented as violin plot: Each point corresponds to one sample; shape shows the frequency distribution of the H‐scores; red line indicates the median of H‐scores; top and bottom lines indicate maximal and minimal values of H‐scores respectively. A total of 23 samples in the Anastrozol arm (18 at diagnostic and 5 on treatment) and 37 samples in the Vistusertib + Anastrozole (29 at diagnostic and 8 on treatment) were analysed.

Using all available samples, total and phosphorylated 4EBP1 and S6K markers were initially evaluated using IHC staining in 47 patients issued from V + A (*n* = 29) and A (*n* = 18) arms, that include all patients for which biopsies were of sufficient amount to perform IHC (Fig. 1A and Fig. S1A). We focused on cytoplasmic S6K and nuclear 4EBP1 stainings as a reflection of mTOR activity. Indeed, while S6K is restricted to the cytoplasm where translation occurs, it has been shown that in response to stress, including in response to the mTOR inhibitor rapamycin, a fraction of 4EBP1 translocates in the nucleus to sequestrate eIF4E that regulates nuclear/cytoplasmic mRNA transport [15]. Comparison of S6K and 4EBP1‐related IHC scores between the two arms at diagnosis showed no drastic difference for these markers as expected (Fig. 1B,C and Fig. S1B,C). When comparing markers at diagnosis and 8 weeks on treatment among the same arm, only a slight increase in phosphorylated S6K and in total nuclear 4EBP1 detection was observed in both V + A and A arms (V + A: diagnosis *n* = 29, on treatment *n* = 8; A: diagnosis *n* = 18, on treatment *n* = 5). In contrast, detection of phosphorylated nuclear 4EBP1 (p4EBP1) was strongly increased in the 8 weeks on treatment biopsies compared to the diagnostic biopsies whatever the arm (Fig. 1C). Such an increase in p4EBP1 between diagnosis and on treatment was also observed among the same patients using diagnosis/on treatment paired samples (V + A, *n* = 8 and A, *n* = 5; Fig. S1D). However, no difference was observed between responders and non‐responders whatever the arm (Fig. S1D). Overall, these data suggest that an increase in nuclear p4EBP1 marker occurs in response to therapy. Since no difference was observed in V + A and A arms, it suggests an association between increased p4EBP1 and Anastrozole treatment.

**Fig. 2:**
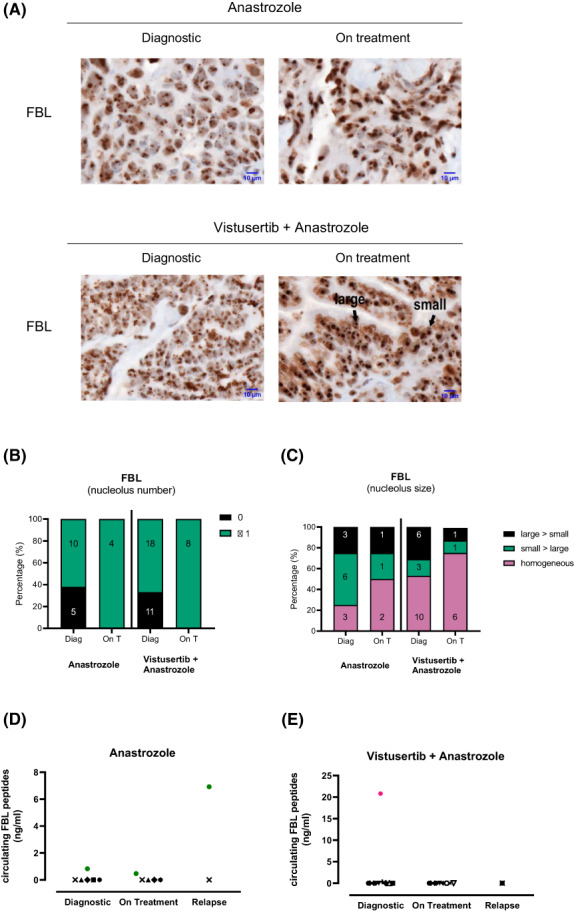
Detection of the ribosome biogenesis factor FBL in biopsies and circulating blood samples. (A–C) detection of fibrillarin (FBL) marker was compared using immunohistochemistry (IHC) between the two arms (A: Anastrozole; V + A: Vistusertib + Anastrozole) and during the course of the patient treatment (Diag: Diagnostic; vs. on T: 8 weeks on treatment). A representative IHC among the four groups for FBL (A, magnification: 90×, scale bar: 10 μm) is given, as well as the score representing the number (B) and the size (C) of FBL dots in the nucleolus. A total of 19 samples in the Anastrozol arm (15 at diagnostic and 4 on treatment) and 37 samples in the Vistusertib + Anastrozole (29 at diagnostic and 8 on treatment) were analysed. (D, E) absolute amount of circulating FBL peptides was quantified in blood samples of six patients treated with Anastrozole (D) and 12 with Vistusertib + Anastrozole (E) during the course of the treatment (diagnostic, 8 weeks on treatment and relapse).

We then analysed two nucleolar RiBi factors, nucleolin (NCL) and fibrillarin (FBL), involved in rRNA synthesis and maturation respectively (Figs 1D–F and 2A–C), as their expression has been associated with poor prognosis in HR+ breast cancer patients [13, 16]. First, both nucleolar and nucleoplasmic NCL scores varied in a similar manner to p4EBP1 (Fig. 1D–F). Indeed, a drastic increase in NCL detection was observed in the on‐treatment biopsies compared to the diagnostic ones, in both arms as well as in responders and non‐responders (Fig. 1D–F and Fig. S1E). Second, when considering FBL, we observed that 16 out of the 44 tumour samples (36%) display no FBL staining at diagnosis (Fig. 2B), supporting our previous observation in breast cancer that some tumours express low level of FBL [16]. Moreover, an increase in the number of dots corresponding to nucleolar FBL staining in the on‐treatment condition compared to diagnosis one was observed in the two arms (Fig. 2A,B), while no clear change was observed in the size of the FBL dots (Fig. 2C). Altogether, these data suggest that increase in p4EBP1 and NCL occurs in on‐treatment compared to diagnostic samples, however, independently of Vistusertib but rather dependent on Anastrozole.

### 3.2 Detection of circulating RiBi‐related peptides in blood samples

To identify putative liquid biomarkers related to RiBi, we analysed autoantibodies. Indeed, detection of circulating RiBi autoantibodies has been reported in several autoimmune diseases but also in serum of cancer patients [17, 18]. Among them, circulating autoantibodies against FBL are used to diagnose systemic scleroderma patients [17, 19]. In a test set of four patients, no circulating FBL autoantibodies was detected using ELISA, at any of the three time points (pre‐treatment, 8 weeks on‐treatment or relapse) or whatever the arm (data not shown).

Detection of circulating FBL peptides have then been evaluated in blood samples at pre‐treatment (*n* = 15), 8 weeks on‐treatment (*n* = 13) and relapse (*n* = 4) using ELISA (Fig. 2D,E). High levels of circulating FBL peptides have been detected at pre‐treatment in one to nine patients in V + A arm, representing one to seven non‐progressive patients (Fig. 2E). In A arm, detection of circulating FBL peptides was observed in one patient that corresponds to the only non‐progressive patient at 8 weeks (Fig. 2D). In this patient, the highest level of circulating FBL peptides occurred at relapse. Surprisingly, these observations suggest that circulating FBL peptides are detectable in blood samples of endometrial cancer patients. Additional studies will be required to determine the use of circulating RiBi‐associated peptides as markers.

### 3.3 mRNA expression of RiBi factors in tumour biopsies according to response to Vistusertib + Anastrozole

**Fig. 3:**
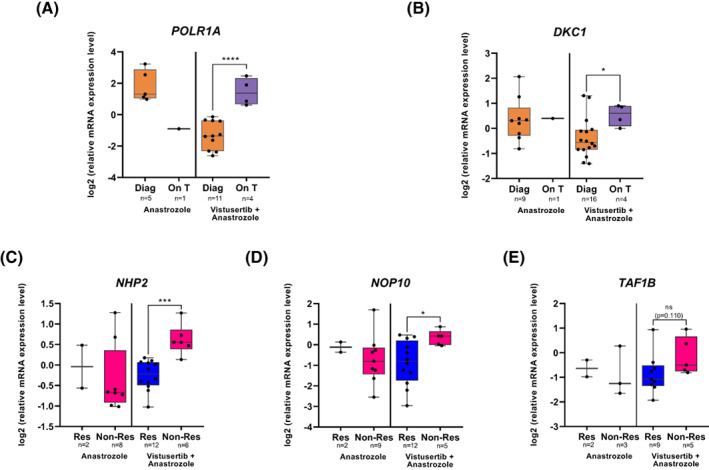
mRNA levels of ribosome biogenesis factors as biomarkers of Vistusertib response. (A, B) comparison of *POLR1A* (A) and *DKC1* (B) mRNA levels between diagnostic (Diag) and 8 weeks on treatment (on T) among the two arms, Anastrozole vs. Vistusertib + Anastrozole. (C–E) comparison of *NHP2* (C), *NOP10* (D) and *TAF1B* (E) mRNA levels between responders (res) and non‐responders (non‐res) among the two arms, Anastrozole vs. Vistusertib + Anastrozole. The number of samples analysed are indicated for each group. Log2 mRNA expression levels are represented as whiskers‐box plot: Each point corresponds to one sample; fill boxes include data between the 25^th^ and the 75^th^ percentile; line within the box indicates the median; whiskers indicate maximal and minimal values (A–E). Wilcoxon–Mann–Whitney non‐parametric test; *P < 0.05; ***P < 0.001. ****P < 0.0001.

Using medium throughput RTqPCR, we analysed the mRNA levels of 21 RiBi genes in 30 patients (V + A, *n* = 18 and A, *n* = 12) for which biopsies were large enough to perform both IHC and RNA analyses. No difference in mRNA levels was observed at diagnosis between the two arms, except for the *RNA polymerase I subunit A* (*POLR1A*) mRNA (Fig. 3A). Comparison of mRNA levels at diagnosis and 8 weeks on‐treatment was performed in both arms, although few samples were available for A arm at on treatment. An increase in mRNA levels was observed for most of the RiBi genes in the V + A arm composed of most of the samples (Fig. S2A,B). An three‐ and twofold increase in *POLR1A* and *Dyskerin pseudouridine synthase 1* (*DKC1*) mRNA levels, respectively, was notably observed at on‐treatment compared to diagnosis (Fig. 3A,B). Such increase was also observed among the paired diagnosis/on‐treatment samples of the same patients (Fig. S2C,D).

We then compared mRNA levels of these 21 RiBi genes at diagnosis between responders and non‐responders. In contrast to the other markers analysed in this study, a difference in mRNA levels was observed in V + A arm but not in A arm for three RiBi genes. In the diagnostic biopsies, drastic and significant increase in mRNA levels of the *NHP2 ribonucleoprotein* (*NHP2*) and *NOP10 ribonucleoprotein* (*NOP10*) was observed in non‐responders (*n* = 6) compared to responders (*n* = 12) in V + A arm (*P* = 0.0002 and *P* = 0.0194, respectively; Fig. 3C,D). In contrast, no difference in *NHP2* and *NOP10* mRNA levels was observed in diagnostic biopsies of non‐responders and responders in A arm, supporting the specificity of *NHP2* and *NOP10* as predictive markers of V + A. A similar increased tendency was observed for the mRNA levels of *TATA‐box binding protein associated factor*, *RNA polymerase I subunit B* (*TAF1B*; Fig. 3E). Altogether, these data indicate that mRNA levels of some RiBi genes might specifically predict response to V + A combination.

To determine whether these three RiBi genes may have a role in determining the outcome of endometrial cancer independently of the specific therapies, we analysed association of their mRNA levels with overall survival using the web portal ualcan (Fig. S3) [14]. Using a series of 543 uterine corpus endometrial carcinoma (UCEC) issued from the TCGA database, a significant association was observed for *NHP2* (*P* = 0.0003) and *TAF1B* (*P* = 0.034), but not for *Nop10* (*P* = 0.77), − high levels of *NHP2* and *TAF1B* being associated with the poorest outcome. Altogether, *NHP2* mRNA levels was not only associated with poor outcome in endometrial cancer independently of the specific therapy in large cohort but also high mRNA levels of *NHP2* in diagnostic biopsies distinguish non‐responders from responders to Vistusertib + Anastrozole combination treatment.

## 4 Discussion

Analysis of the ribosome biogenesis factors' expression in the VICTORIA clinical trial identifies a set of preliminarily RiBi‐based markers to monitor drug activity but also to predict the response to Anastrozole and mTOR inhibitor (Vistusertib) combination therapy in advanced endometrial cancer.

Increase in RiBi markers was paralleled with endocrine therapy on treatment, suggesting we identified novel markers of Anastrozole activity, when used either alone or in combination. Indeed, an increase in p4EBP1 nuclear staining, but not of total 4EBP1 nuclear staining, was observed at 8 weeks of treatment compared to a diagnosis in both A and V + A arm, suggesting a mTOR inhibitor‐independent effect. Similar observation was done for NCL staining, suggesting that this multifunctional protein is affected by Anastrozole [20]. Whether the NCL marker is affected by other aromatase inhibitors remains to be investigated. Moreover, a global increase in RiBi mRNA levels was observed in both A and V + A arm. This increase in RiBi factors at both mRNA and protein levels is consistent with previous study demonstrating that tamoxifen promotes expression of RiBi factors [21]. Interestingly, increase in circulating FBL peptides was also observed in one non‐progressive patient treated with Anastrozole only. Such observation might result from an increase in tumoural cell death releasing tumoural‐associated peptides. It would be of interest to analyse circulating exosomal *FBL* mRNA, as an additional RiBi‐associated circulating biomarker. Thus, further evaluation might reveal new RiBi‐associated biomarkers of Anastrozole activity, including circulating ones.

Usage of Vistusertib was expected to decrease expression of RiBi factors since mTOR is one of the main coordinators of RiBi [8, 10]. In contrast, no decrease in RiBi was observed in response to mTORC1 inhibitor for most markers. We supposed that Anastrozole and mTORC1 inhibition by Vistusertib could be insufficient to circumvent the effect of Anastrozole that probably induced high levels of RiBi.

In the VICTORIA trial reporting positive results for aromatase/mTOR inhibitors, it is of importance to identify potential biomarkers of efficacy for Vistusertib + Anastrozole combination therapy for future clinical trials. We identified potential predictive biomarkers of response (i.e. 8 weeks progression‐free survival as endpoint) among the pathway of ribosome biogenesis. mRNA levels of *NHP2* and *NOP10* indeed specifically distinguished non‐responders from responders to the Vistusertib + Anastrozole combination. Interestingly, it has been shown that *NHP2* expression level was associated with prognosis in endometrial cancer [22]. Moreover, we also observed in a large dataset that high level of *NHP2* was associated with poor outcome, in consistence with the fact that high *NHP2* levels predict the lack of response to the Vistusertib + Anastrozole combination. *NHP2* and *NOP10* genes encode proteins that are components of the H/ACA box snoRNPs along with the pseudouridine synthase DKC1, which regulates the pseudo‐uridylation of rRNA [23]. Knowing the emerging concept of specialized ribosomes suggesting that change in ribosome composition, including in rRNA pseudouridylation, might affect ribosome activity to specify a particular mRNA translational program, it would be of interest in the near future to investigate the impact of mTORC1 inhibitors on ribosome composition [24, 25, 26, 27]. Other RiBi factors might be of interest as biomarker of efficacy, including NOL6, the human homologous of a yeast snoRNA‐associated protein complex involved in rRNA maturation that has been shown to promote proliferative and migration capacities of endometrial cancer cells [28].

Overall, these RiBi factors might correspond to the first RiBi‐associated predictive markers of aromatase and mTOR inhibitor combination therapy at least in advanced stages of endometrial cancer. It must be noted that our data rely on only a small number of samples. Thus, these preliminary observations need to be reproduced in larger cohorts.

## 5 Conclusions

Altogether, our preliminary evaluation highlights the potential usage of RiBi factors as innovative biomarkers of drug activity and of prediction of treatment outcome in cancer. In particular, *NHP2* is the most promising one. These predictive markers could be easily analysed by classical RTqPCR and development of IHC staining will allow their future evaluation in clinical trials to assess their usage in improving cancer patient management through a better estimation of the most appropriate therapeutic strategy.

## Conflict of interest

The authors declare no conflict of interest.

## Author contributions

N‐E‐HM, AC‐V, LO, VS, IT performed and analysed experiments. CD, SC, DP performed statistical description. J‐SF, MF, SM, ST‐E, IR‐C, FB, SA‐L, EC provided human samples. GG coordinated the samples and data workflow. N‐E‐HM, CD, IT, SC, GG, P‐EH, J‐JD, VM interpreted the data. P‐EH, J‐JD, VM shaped the clinical and research question and supervised the project coordination. N‐E‐HM and VM wrote the first draft of the manuscript. All authors have read and approved the manuscript.

### Peer review

The peer review history for this article is available at https://publons.com/publon/10.1002/1878‐0261.13340.

## Supporting information


**Fig. S1.** Changes of mTOR targets and NCL staining according to aromatase and mTOR inhibitors treatment.

**Fig. S2.** mRNA levels of 21 RiBi factors in response to Anastrozole and Vistusertib + Anastrozole treatments.

**Fig. S3.** Association of mRNA levels of 3 RiBi factors with patient outcome in endometrial cancer independently of treatment.

**Table S1.** Antibodies and scores for immunohistochemistry (IHC) staining.
**Table S2.** Multiplex PCR and qPCR primers sequence.

## Data Availability

The data that support the findings of this study are available on request from the corresponding author. The data are not publicly available due to privacy or ethical restrictions.
