# Supplementary figures and images for: Ribosome biogenesis‐based predictive biomarkers in endocrine therapy (Anastrozole) combined with mTOR inhibitor (Vistusertib) in endometrial cancer: translational study from the VICTORIA trial in collaboration with the GINECO group
Source: Mol Oncol. 2022 Dec 7;17(1):27–36. doi: 10.1002/1878-0261.13340 (PMC9812831; doi:10.1002/1878-0261.13340)

# Figure S1

(A)

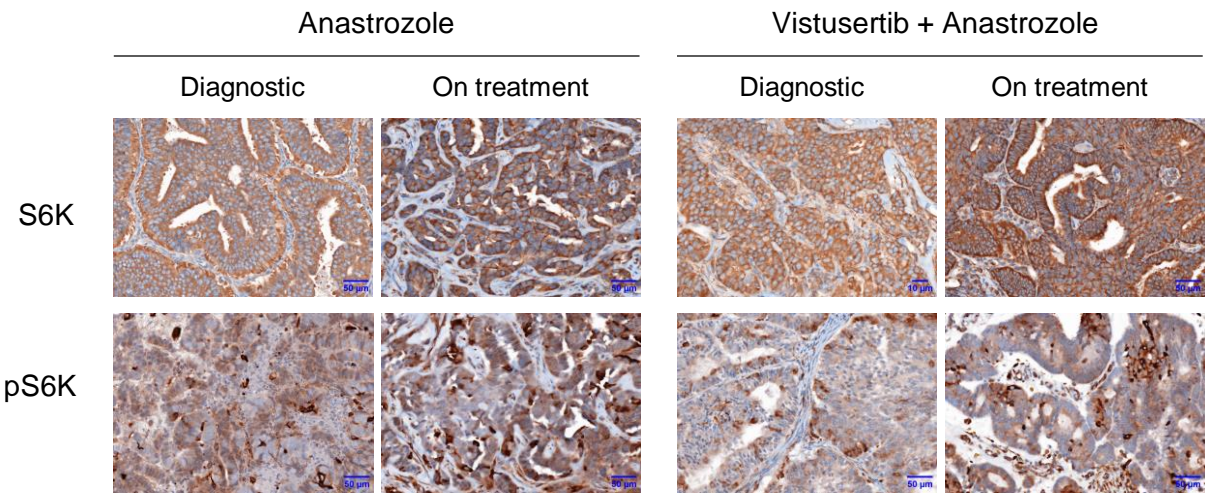

(B)

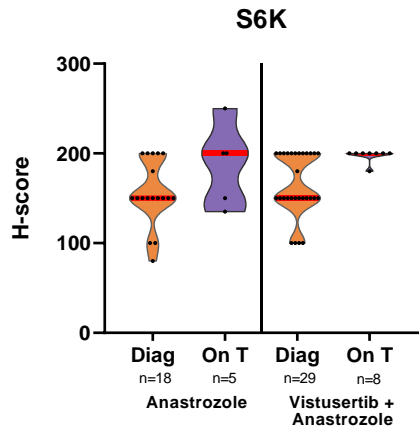

(C)

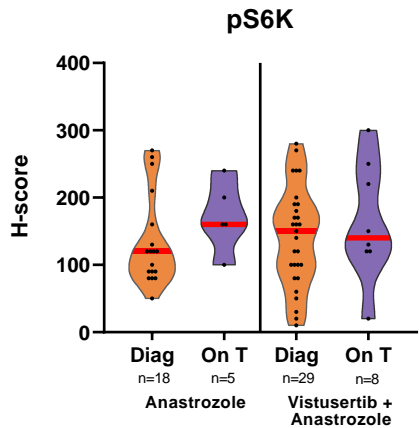

(D)

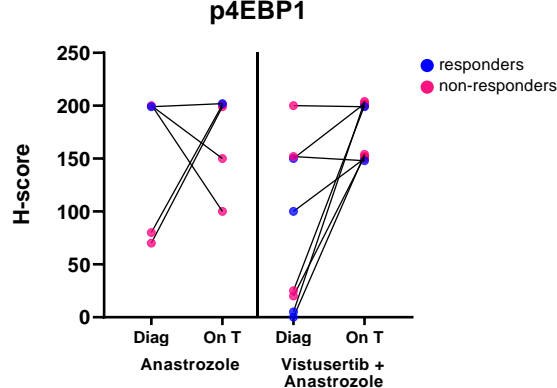

(E)

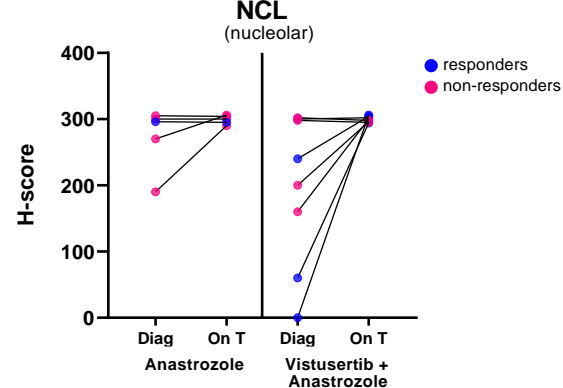

Supplement: Supplementary file 1 — Fig. S1. Changes of mTOR targets and NCL staining according to aromatase and mTOR inhibitors treatment. [file MOL2-17-27-s003.pdf]

Figure S2

(A)

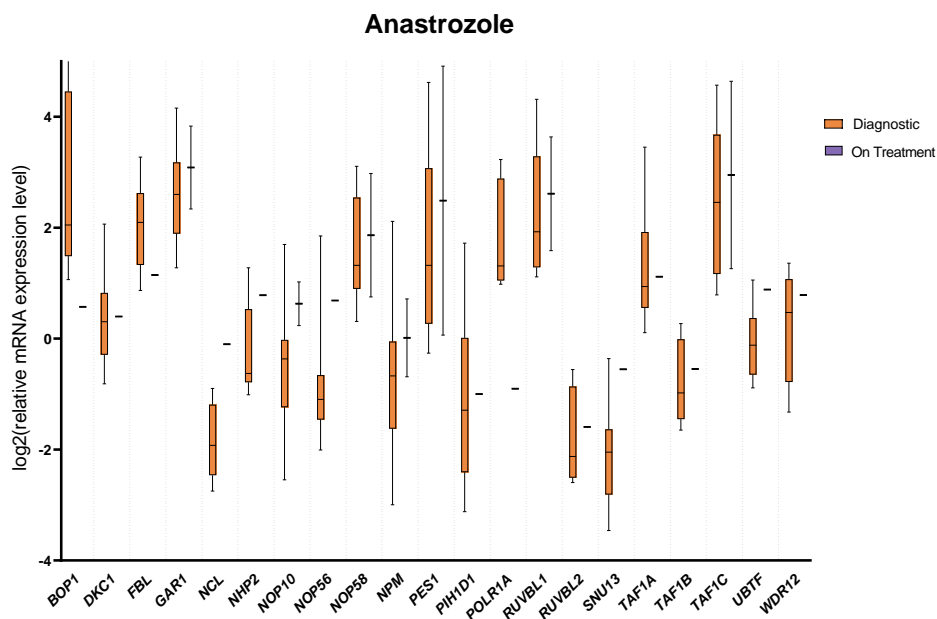

(B)

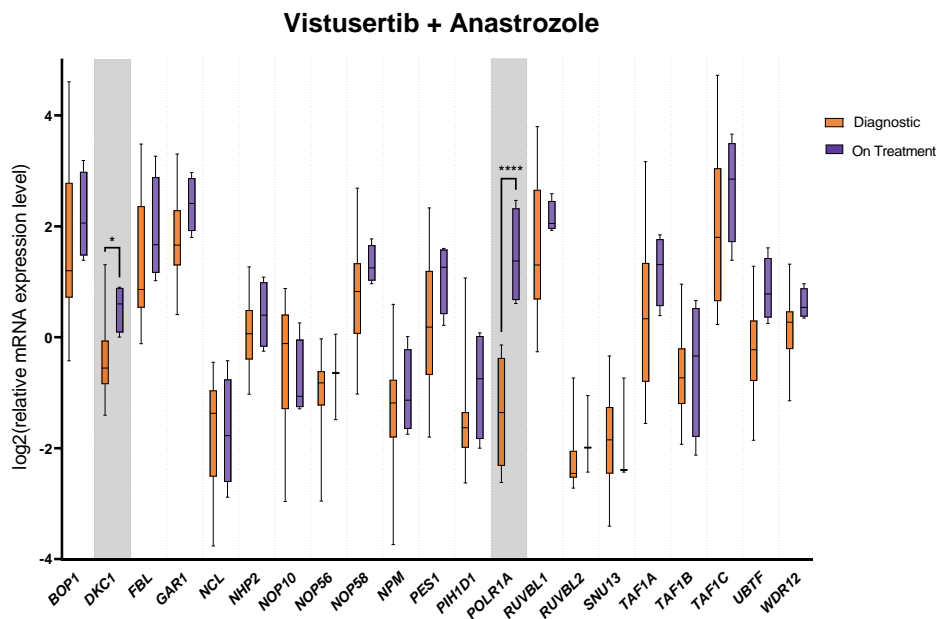

(C)

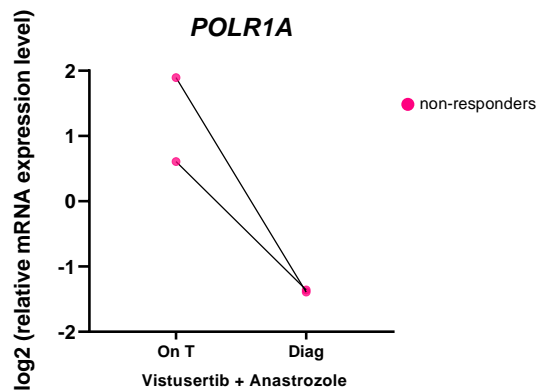

(D)

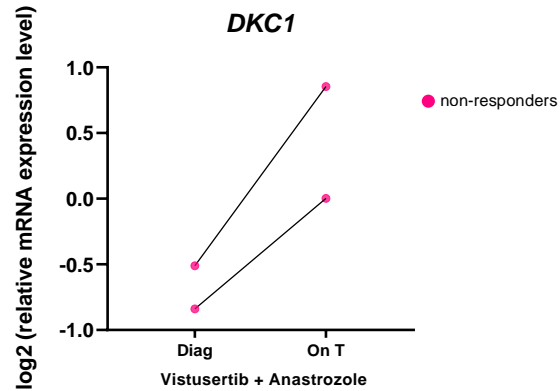

Supplement: Supplementary file 2 — Fig. S2. mRNA levels of 21 RiBi factors in response to Anastrozole and Vistusertib + Anastrozole treatments. [file MOL2-17-27-s002.pdf]

(A)

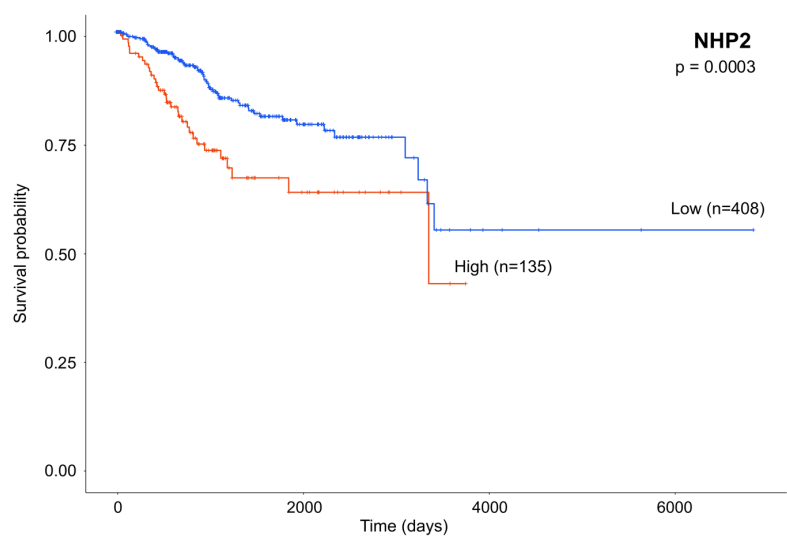

(B)

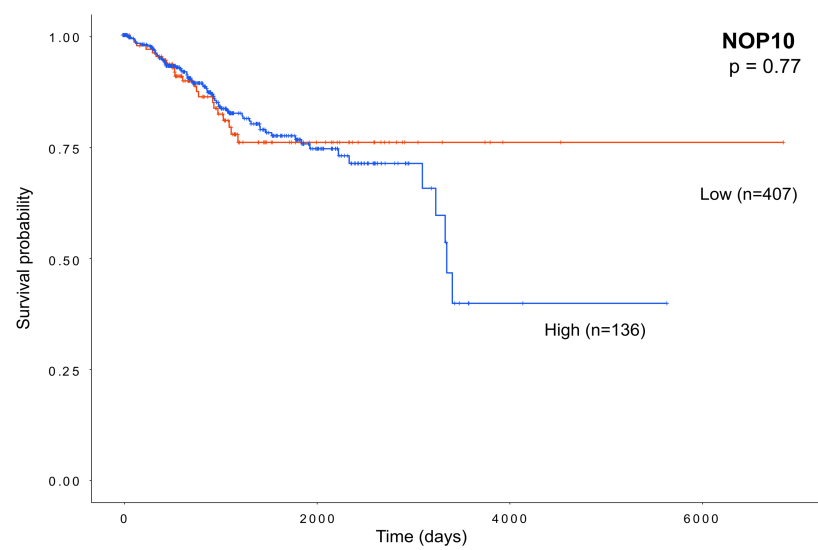

(C)

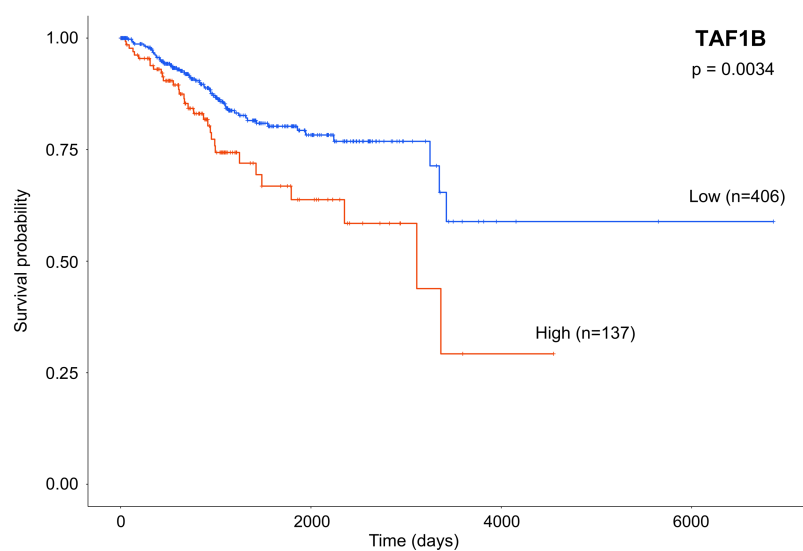

Supplement: Supplementary file 3 — Fig. S3. Association of mRNA levels of 3 RiBi factors with patient outcome in endometrial cancer independently of treatment. [file MOL2-17-27-s004.pdf]
