# Supplementary material for: Ribosome biogenesis‐based predictive biomarkers in endocrine therapy (Anastrozole) combined with mTOR inhibitor (Vistusertib) in endometrial cancer: translational study from the VICTORIA trial in collaboration with the GINECO group
Source: Mol Oncol. 2022 Dec 7;17(1):27–36. doi: 10.1002/1878-0261.13340 (PMC9812831; doi:10.1002/1878-0261.13340)
Supplement: Supplementary file 4 — Table S1. Antibodies and scores for immunohistochemistry (IHC) staining. Table S2. Multiplex PCR and qPCR primers sequence. [file MOL2-17-27-s001.docx]

**Ribosome biogenesis-based predictive biomarkers in endocrine therapy (Anastrozole) combined with mTOR inhibitor (Vistusertib) in endometrial cancer: translational study from the VICTORIA trial in collaboration with the GINECO group**

Nour-El-Houda Mourksi^1,2,3^, Cécile Dalban^4^, Amélie Colombe-Vermorel^5^, Laetitia Odeyer^5^, Valentin Simioni^1,2,3^, Jean-Sébastien Frenel^6^, Michel Fabbro^7^, Fernando Bazan^8^, Sophie Abadie-Lacourtoisie^9^, Elodie Coquan^10^, Séverine Martinez^11^, Gwenaelle Garin^4^, Séverine Tabone-Eglinger^11^, Isabelle Treilleux^5^, Sylvie Chabaud^4^, David Pérol^4^, Isabelle Ray-Coquard^12^, Pierre-Etienne Heudel^12^, Jean-Jacques Diaz^1,2,3*^, Virginie Marcel^1,2,3*^

^1^Centre de Recherche en Cancérologie de Lyon, Inserm U1052, CNRS UMR5286, Université de Lyon, Université Claude Bernard Lyon 1, Centre Léon Bérard, CEDEX 08, F-69373 Lyon, France

^2^Institut Convergence PLAsCAN, Lyon, France

^3^DevWeCan Labex Laboratory, Lyon, France

^4^Clinical Research Department, Centre Léon Bérard, Lyon, France

^5^Biopathology Department, Centre Léon Bérard, GINECO, Lyon, France

^6^Medical Oncology Department, Institut Cancérologie de l’Ouest, and GINEGEPS, St Herblain, France

^7^Department of Surgical Oncology, Institut du Cancer de Montpellier, University of Montpellier, Montpellier, France

^8^Department of Medical Oncology, University Hospital of Besançon, Besançon, France
^9^Institut de Cancérologie de l'Ouest, Angers, France

^10^Department of Clinical research, Department of Medical Oncology, Comprehensive Cancer Centre François Baclesse, Caen, France

^11^Biological Ressource Center, Centre Léon Bérard, Lyon, France

^12^Medical Oncology Department, Centre Léon Bérard and University Claude Bernard Lyon 1, GINECO, Lyon, France

*Corresponding authors: [virginie.marcel@lyon.unicancer.fr](mailto:virginie.marcel@lyon.unicancer.fr) and [jean-jacques.diaz@lyon.unicancer.fr](mailto:jean-jacques.diaz@lyon.unicancer.fr)

**Supplementary Tables p2-3**

**Supplementary Figure Legends p4**

**Supplementary Table S1. Antibodies and scores for immunohistochemistry (IHC) staining**

| Marker | Brand | Reference | Dilution | IHC score |
| --- | --- | --- | --- | --- |
| S6K | Cell Signaling | 2317 | 1/25 | % stained tumoral cells x intensity of cytoplasmic staining |
| pS6K | Cell Signaling | 4857 | 1/100 | ∑(% stained tumoral cells x i) [i = intensity of cytoplasmic staining groups, from 1 to 3] |
| 4EBP1 | Cell Signaling | 9644 | 1/1500 | % stained tumoral cells x intensity of cytoplasmic staining |
| p4EBP1 | Cell Signaling | 9451 | 1/100 | % stained tumoral cells x intensity of cytoplasmic staining |
| NCL | Enzo Life Sciences | ADI-KAM-CP100 | 1/2000 | % stained tumoral cells x intensity of cytoplasmic staining |
| FBL | Abcam | ab154806 | 1/300 | Number of nucleoli: 0 or ≥1  Size of nucleoli: 0 = similar size, 1 = number of small nucleoli > number of large nucleoli, 2 = number of small nucleoli < number of large nucleoli |

**Supplementary Table S2. Multiplex PCR and qPCR primers sequence**

| Genes | Forward primer | Reverse primer |
| --- | --- | --- |
| **Actin* | 5’-CCAACCGCGAGAAGATGA-3’ | 5’-TCCATCACGATGCCAGTG-3’ |
| *BOP1* | 5’-GACGATCCTGACTACTGGCG-3’ | 5’-ACCTGCTCATCCGTCAGTCT-3’ |
| *DKC1* | 5’-CCCTTTGGAAAAGCTGTTGA-3’ | 5’-TAATCTTGGCCCCATAGCAG-3’ |
| *FBL #1* | 5’-CCTGGGGAATCAGTTTATGG-3’ | 5’-CCAGGCTCGGTACTCAATTTT-3’ |
| *FBL #2* | 5’-CCTGCGTAATGGAGGACACT-3’ | 5’-GCTGAGGCTGTGGAGTCAAT-3’ |
| **GAPDH* | 5’-AGCCACATCGCTCAGACAC-3’ | 5’-GCCCAATACGACCAAATCC-3’ |
| *GAR1* | 5’-CAAGACCAAGGACCTCCAGA-3’ | 5’-TCATCTTCACAGGGATGCAG-3’ |
| **HPRT1* | 5’-TGACACTGGCAAAACAATGCA-3’ | 5’-GGTCCTTTTCACCAGCAAGCT-3’ |
| *NCL #1* | 5’-CCAGAACCAAAATGGCAAAT-3’ | 5’-CTGATTGCTCTGCCCTCAAT-3’ |
| *NCL #2* | 5’-GTCAGCAAGGATGGGAAAAG-3’ | 5’-TAGATCGCCCATCGATCTCT-3’ |
| *NHP2* | 5’-GGTCAACCAGAACCCCATC-3’ | 5’-TTCTGCTTCACCGCTTTCTT-3’ |
| *NOP10* | 5’-GAAGAAATTTGACCCGATGG-3’ | 5’-TGAAGCGTTTCTTGATGGTG-3’ |
| *NOP56* | 5’-AGGCTATTCTGGATGCCTCA-3’ | 5’-GTAGGCTCTGGCGGTATTCA-3’ |
| *NOP58* | 5’-TGATGGAGGGCAAAATCAAT-3’ | 5’-CGGTTCATGGGCTTCTTTTA-3’ |
| *NPM* | 5’-TTGTTGAAGCAGAGGCAATG-3’ | 5’-TATTTCAAAGCCCCCAAGG-3’ |
| *PES1* | 5’-GGCAAGAGGCGAAAAATCCG-3’ | 5’-TTCTTCTCAGACCTCACCGC-3’ |
| **PGK1* | 5’-AAGTGAAGCTCGGAAAGCTTCTAT-3’ | 5’-AGGGAAAAGATGCTTCTGGG-3’ |
| *PIH1D1* | 5’-TGAGTCTGGGAGAGCCTCAT-3’ | 5’-AAATCGCTGTTCTGCATCCT-3’ |
| *POLR1A* | 5’-CTGAGCCCCTGGGAATTGAG-3’ | 5’-CCTTCATTCTTCCACAGGGCA-3’ |
| **PPIA* | 5’-GTCAACCCCACCGTGTTCTT-3’ | 5’-CTGCTGTCTTTGGGACCTTGT-3’ |
| *RUVBL1* | 5’-AAGGGGATGTGC ACA AAA AG | 5’-CACATCCAAGTCATGCAAGG-3’ |
| *RUVBL2* | 5’-CGCTCTTCTCAGGTGACACA-3’ | 5’-CAGCACTCCAGGGATGATCT-3’ |
| *SNU13* | 5’-GCTACTGGACCTCGTTCAGC-3’ | 5’-ACTCAGAGATGCCCCTGTTG-3’ |
| *TAF1A* | 5’-TCCTGGAGTTTGGGACCCTT-3’ | 5’-TGGTGAGTACCTCTTGGGCT-3’ |
| *TAF1B* | 5’-CGAGGAGGCGGAAGAGTTTA-3’ | 5’-TCTCTCTGTAACATTGTGGCAAGA-3’ |
| *TAF1C* | 5’-CGGAGTGAAGATGCTGGACA-3’ | 5’-GCCCCCAAACGAAAAAGCAA-3’ |
| *UBTF* | 5’-TGTGGAACGACCTGTCTGAG-3’ | 5’-CTCTCCGACTGAGCCTTGAG-3’ |
| *WDR12* | 5’-TAAAGGGGCAGAGGAATGGAT-3’ | 5’-CAACATCCGTATGTCCCACAA-3’ |

********Housekeeping genes*

**Supplementary Figure Legends**

**Supplementary Figure 1. Changes of mTOR targets and NCL staining according to aromatase and mTOR inhibitors treatment. (A-C)** Detection of total and phosphorylated S6K marker (S6K and pS6K, respectively) was compared using immunohistochemistry (IHC) between the two arms (A: Anastrozole; V+A: Vistusertib+Anastrozole) and during the course of the patient treatment (Diag: diagnostic; vs On T: 8-weeks on treatment). A representative IHC is given among the four groups for S6K (A, magnification: 30x, scale bar: 50µm). H-scores of total (B) and phosphorylated S6K (C) stainings are represented as violin plot: each point corresponds to one sample; shape shows the frequency distribution of the H-scores; red line indicates the median of H-scores; top and bottom lines indicate maximal and minimal values of H-scores, respectively (B,C). A total of 23 samples in the Anastrozole arm (18 at diagnostic and 5 on treatment) and 37 samples in the Vistusertib+Anastrozole (29 at diagnostic and 8 on treatment) were analyzed. **(D-E)** Evolution of p4EBP1 marker (D) and nucleolar NCL marker (E) in paired diagnostic/on-treatment samples for responder and non-responder patients is represented for Anastrozole arm (n=5) and Vistusertib+Anastrozole arm (n=8).

**Supplementary Figure 2. mRNA levels of 21 ribosome biogenesis factors in response to Anastrozole and Vistusertib+Anastrozole treatments. (A-B)** Comparison of the mRNA levels of 21 ribosome biogenesis (RiBi) factors between diagnostic (orange) and 8-weeks on treatment (violet) among the two arms, Anastrozole (A) vs Vistusertib+Anastrozole (B). Log2 mRNA expression levels are represented as whiskers-box plot: each point corresponds to one sample; fill boxes include data between the 25^th^ and the 75^th^ percentile; line within the box indicates the median; whiskers indicate maximal and minimal values. Wilcoxon-Mann-Whitney non-parametric test; *: P<0.05; ****: P<0.0001; significant changes are highlighted in gray. **(C-D)** Evolution of *POLR1A* (D) and *DKC1* (E) mRNA levels in paired diagnostic (Diag)/8-weeks on-treatment (On T) samples for some responder and non-responder patients are represented for Anastrozole arm (n=2) and Vistusertib+Anastrozole arm (n=2).

**Supplementary Figure 3. Association of mRNA levels of 3 ribosome biogenesis factors with patient outcome in endometrial cancer independently of treatment. (A-C)** Using the web portal UALCAN, association between *NHP2* (A), *NOP10* (B) or *TAF1B* (C) mRNA levels and overall survival (OS) was determined using log-rank tests generated using the Kaplan-Meier method in TCGA datasets (n = 543). OS is represented as Kaplan-Meier curves, the proportion of patients being given depending on the timing from date of diagnosis to either death from all causes or last follow-up for censored patients (vertical line). The number of samples analyzed are indicated for each group.
